# A Bullet Entered through the Open Mouth and Ended Up in the Parapharyngeal Space and Skull Base

**DOI:** 10.1155/2015/680279

**Published:** 2015-06-07

**Authors:** Saileswar Goswami, Choitali Goswami

**Affiliations:** ^1^Department of Otolaryngology, Calcutta National Medical College, Kolkata, West Bengal 700014, India; ^2^Department of General Emergency, Medical College, Kolkata, West Bengal 700073, India

## Abstract

Shot from a revolver from a close range, a bullet pierced the chest of a policeman and entered through the open mouth of a young male person standing behind. The entry wound was found in the cheek mucosa adjacent to the left lower third molar. After hitting and fracturing the body and the ramus of the mandible, the bullet was deflected and was finally lodged in the parapharyngeal space and skull base, anterolateral to the transverse process of the atlas. The great vessels of the neck were not injured. The patient's condition was very critical but his life could be saved. The bullet was approached through a modified Blair's incision and was found to be lying over the carotid sheath. It was removed safely and the patient recovered completely.

## 1. Introduction

Foreign bodies in the parapharyngeal spaces are rare. Bora et al. [1] reported a case of bullet in the left parapharyngeal space at the level of C2–C4. In the present case, a bullet entered through the open mouth and after breaking the angle and ramus of the mandible on the left side, it traversed through the soft tissue of the neck. It was finally lodged in the left parapharyngeal space and skull base, just anterolateral to the transverse process of the atlas. It was surprising that the great vessels of the neck were spared and the patient survived. The reason behind reporting this case was its extreme rarity and the challenge it posed in its safe removal.

## 2. Case Presentation

In the evening, a 32-year-old man and two policemen were trying to pacify another man in a case of domestic violence. The accused took out the revolver of one of the policemen and shot him on the left side of his chest. The bullet came out through the back of the policeman and entered through the open mouth of the patient, who was standing behind him. The policeman died on the way to the hospital. The patient became unconscious and was admitted to the nearby hospital. After initial treatment, the patient was referred to a Medical College and was admitted there. He was given supportive treatment including blood transfusion and he regained his consciousness in the next morning.

**Figure 1:**
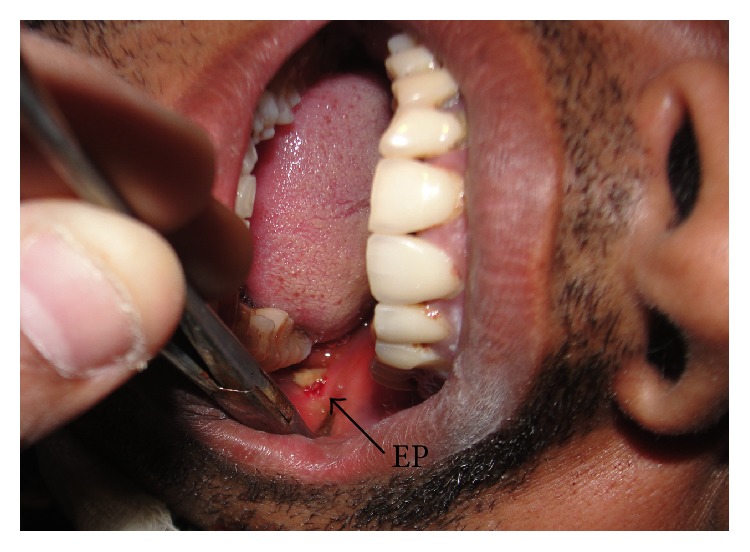
Preoperative photograph showing the entry point (EP) of the bullet (1.2 cm × 1.0 cm) on the cheek mucosa adjacent to the left lower third molar.

On examination, there was a huge haematoma over the left side of the face. The entry wound (Figure 1) was found in the inner aspect of the cheek adjacent to the left lower third molar. There was dental malocclusion. A fracture line could be palpated near the angle of the mandible on the left side. The patient was unable to open his mouth completely.

There was no injury in the pharynx. Airway was clear. There was an area of bruise with extra tenderness over the left infra-auricular area. On bimanual palpation, the patient complained of sharp pain and was able to feel the bullet there. The cranial nerves were normal.

**Figure 2:**
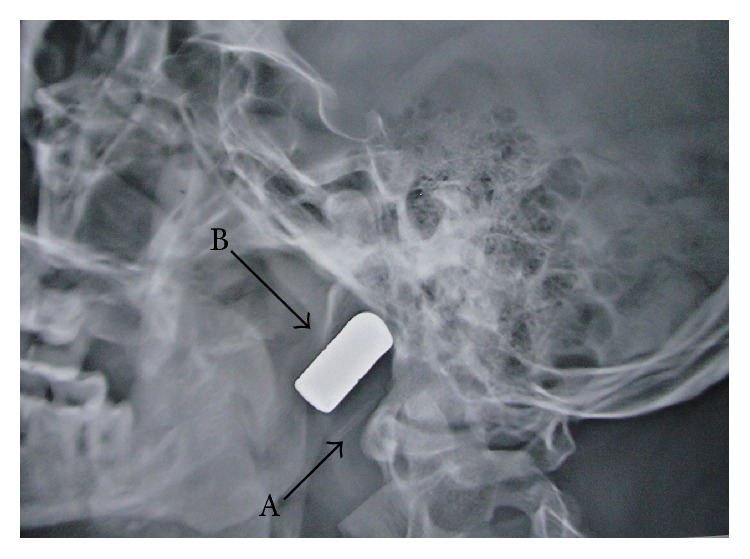
Preoperative X-ray of the skull, lateral view, showing the bullet (B) in the left parapharyngeal space at the level of the atlas (A).

**Figure 3:**
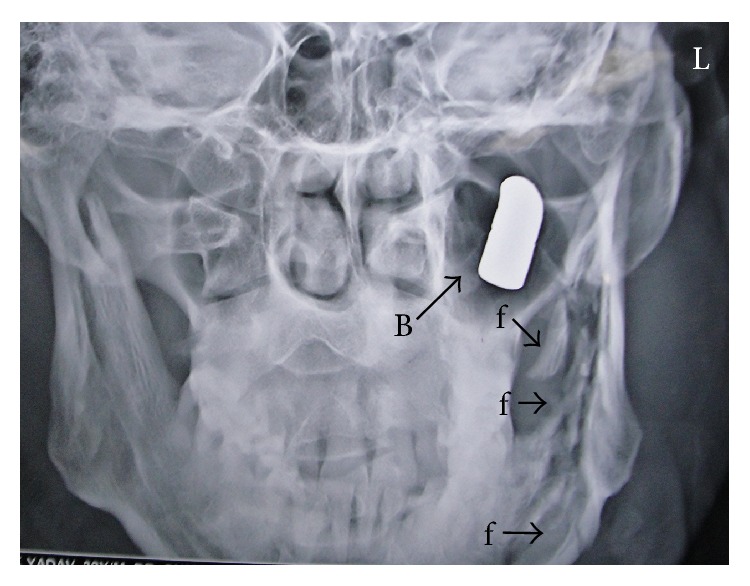
Preoperative X-ray of the skull, anteroposterior view, showing the bullet (B) lying in a sagittal plane passing through the middle of the left maxillary antrum. Note the multiple fracture fragments (f) of the mandible on the left side.

Lateral view of the X-ray of the skull (Figure 2) revealed a bullet in the base of the skull on the left side at the level of the atlas. It was placed obliquely occupying an area posterior to the neck of the mandible, extending from the level of the atlas to the basiocciput. The base was directed anteroinferiorly and the tip was directed posterosuperiorly. Anteroposterior view of the X-ray of the skull (Figure 3) revealed that it was lying in a sagittal plane passing through the middle of the left maxillary sinus. The body and ramus of the mandible were broken near its left angle with multiple fracture fragments.

**Figure 4:**
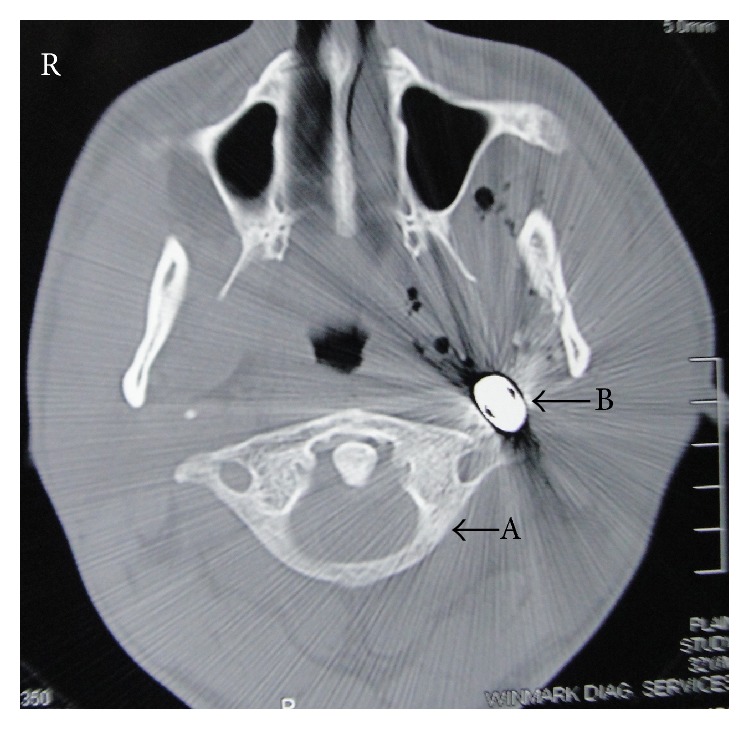
CT scan passing through the atlas, showing the bullet (B) in the left parapharyngeal space anterolateral to the transverse process of the atlas (A) with air pockets around it. Note the reflection of X-rays by the bullet.

**Figure 5:**
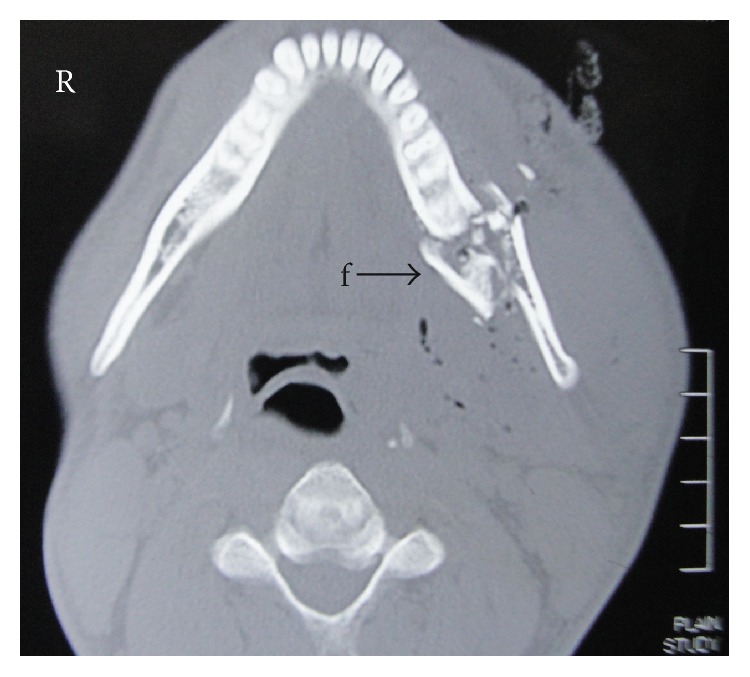
Axial CT scan showing multiple fractures of the mandible with inward displacement.

CT scan revealed the bullet in the parapharyngeal space (Figure 4) about one cm anterolateral to the transverse process of the atlas on the left side. There was diffused soft tissue thickening adjacent to the foreign body with multiple air pockets, extending up to the left malar region. There were multiple fractures involving the ramus and the body of the mandible on the left side with inward displacement of the fragments (Figure 5). The bullet was found to be in close proximity to the vertebral artery and the carotid sheath. Due to reflection of the X-ray by the bullet, the image was blurred and it was not possible to assess soft tissue details.

The initial plan was to remove the bullet and manage the fractures of the mandible at the same time. However, the faciomaxillary surgeons preferred to deal with the fractures of the mandible in a second stage. The treatment plan was modified accordingly and the patient was prepared for removal of the bullet through an external approach.

**Figure 6:**
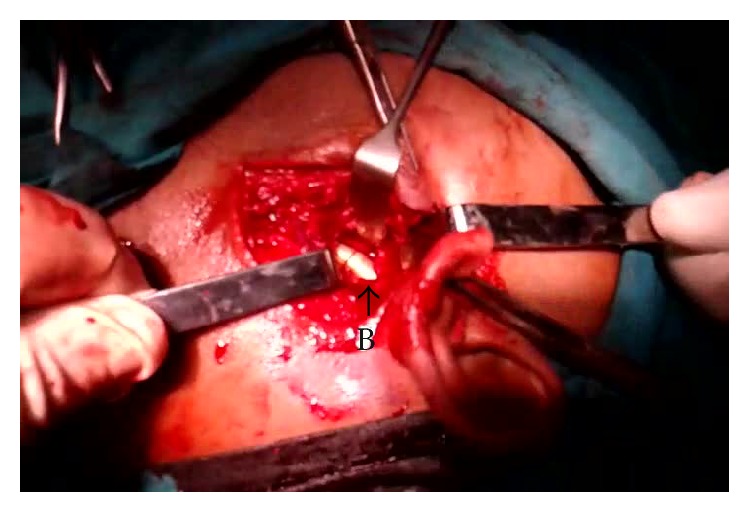
Peroperative photograph, showing the bullet (B) in the left parapharyngeal space with its tip directed posterosuperiorly.

**Figure 7:**
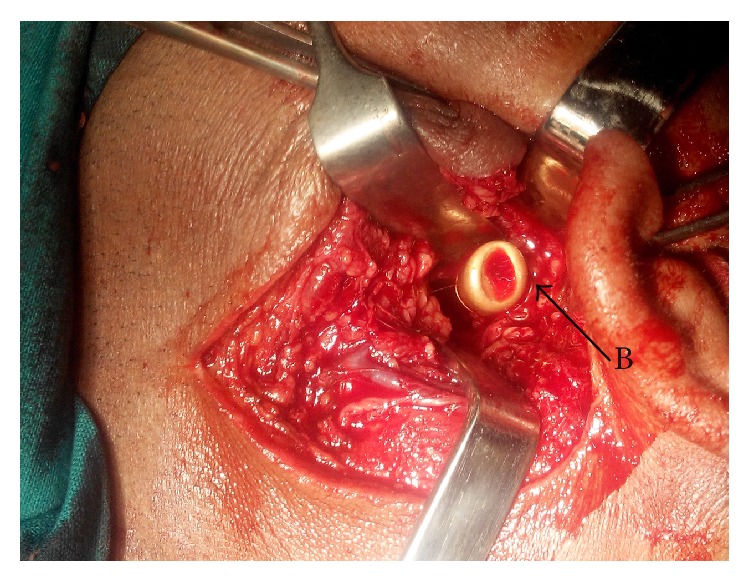
Peroperative photograph, showing the bullet (B) in the left parapharyngeal space after mobilisation of its tip.

**Figure 8:**
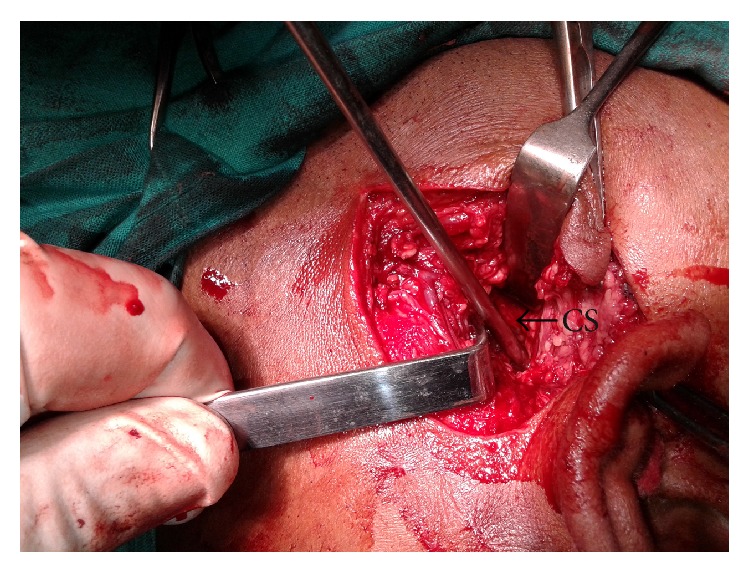
Peroperative photograph of the cavity created by the bullet with exposure of the carotid sheath (CS).

**Figure 9:**
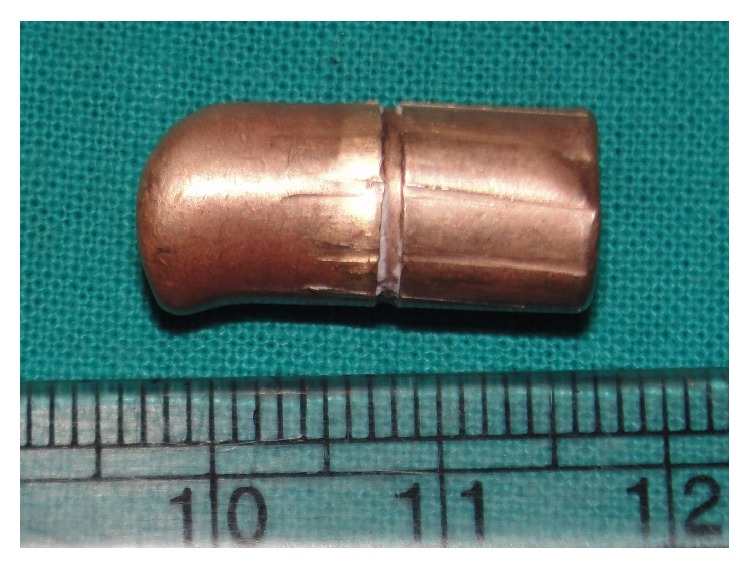
The bullet after removal (2 cm × 0.9 cm).

**Figure 10:**
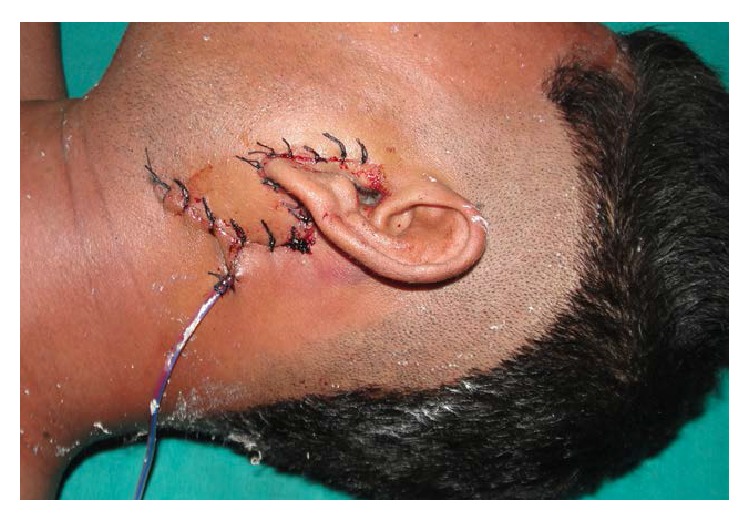
Photograph of the surgical area on the 1st postoperative day.

The approach was made through a modified Blair's incision as in parotidectomy. The flap was elevated at the subplatysmal level. The main trunk of the facial nerve was identified first. The parotid gland along with the ramus of the mandible was retracted anterosuperiorly and the sternomastoid muscle was retracted posteriorly. Blunt dissection was carried out very carefully up to the carotid sheath but the bullet could not be found. Bimanual palpation was done with the left index finger in the dissected area and the right middle finger in the pharynx. The bullet could be felt between the fingers. Dissection was continued further medially. Finally, the bullet was found lying obliquely with its tip directed posterosuperiorly (Figure 6). It was touching the posterior surface of the carotid sheath and was pulsating with it. The tip of the bullet was mobilized from the surrounding tissue gently with the help of a blunt square hook. Surprisingly, it was very easy to free the tip as the bullet was surrounded by a cushion of air. The direction of its tip was changed outwards and upwards (Figure 7). Then, the bullet was removed by holding it with an artery forceps. The carotid sheath was found to be exposed but intact (Figure 8). Residual necrotic tissue was removed and the area was repeatedly washed with normal saline to clean the dirt. A mini-vacuum drain was placed inside and the wound was closed in layers. The bullet was measured and was found to be 2 cm long and 0.9 cm in diameter (Figure 9). The patient recovered quickly (Figure 10) and was discharged on the eighth postoperative day.

**Figure 11:**
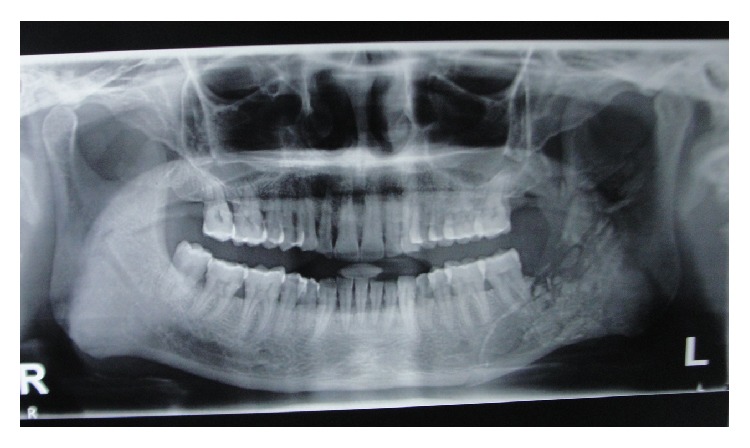
Orthopantomogram of the jaws three weeks after the injury.

**Figure 12:**
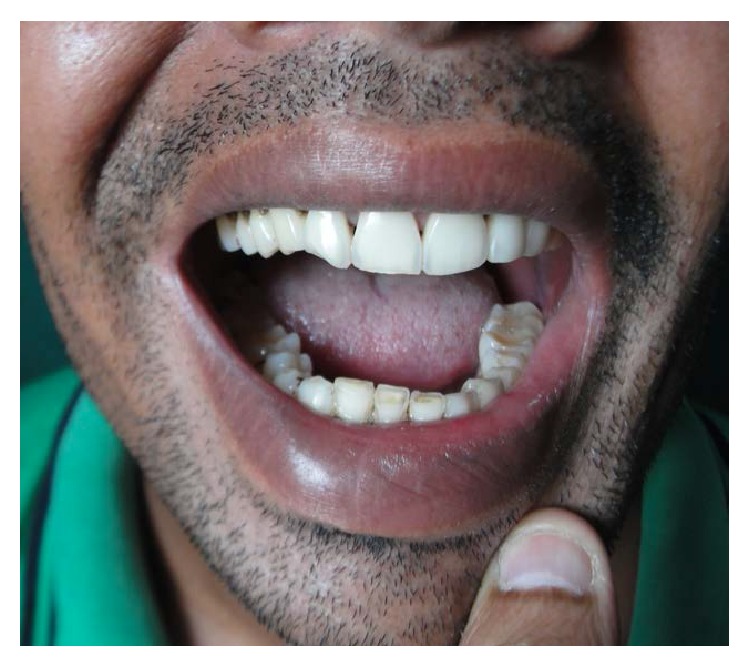
Photograph of the teeth of the patient six weeks after the injury.

On subsequent follow-ups, the patient's condition was found to be very good. All the wounds had healed nicely. The only problem he was having was mild difficulty in chewing, which was due to the fractures of the mandible. After going through the traumatic experience, the patient was scared and was unwilling to undergo another operation. That problem of chewing also started to be relieved gradually. OPG done three weeks after the incident (Figure 11) revealed that the fractures of the mandible had started healing. After six weeks, the patient was able to chew solid foods. Although the patient was having slight malocclusion, he was not having much problem in taking his regular diet (Figure 12).

## 3. Discussion

Foreign bodies in the parapharyngeal spaces are not common. There are reports of foreign bodies like broken toothbrush, [2] glass fragments [3, 4], and pen [5] in the parapharyngeal space. Goswami [6] reported a case of bullet in the maxillary antrum and infratemporal fossa.

Radio-opaque foreign bodies in the head and neck can easily be detected by plain X-ray. CT scan and MRI are necessary to assess the actual position of the foreign bodies. Angiogram and colour Doppler study are helpful in cases of foreign bodies situated close to important vessels.

Foreign bodies in the head and neck are difficult to manage due to the presence of the great vessels, important nerves, and other vital structures. Initial management should be concentrated upon securing the airway and controlling the bleeding. In case of injury to the aerodigestive tract or major neurovascular structures, the foreign body should be removed at the earliest.

There are controversies about the time of removal of foreign bodies without any significant injury to the vital structures. A retained foreign body can lead to chronic infection, cutaneous fistula, and foreign body granuloma. It can even migrate through the common carotid artery [7] or internal jugular vein [8]. In case of a bullet, there may be lead poisoning [9]. There is a possibility of carotid artery blow-up if the bullet is situated near it.

In the present case, there was no injury in the aerodigestive tract. The patient could be taken out of the initial crisis successfully. However, as the bullet was lodged in a potentially dangerous area, it had to be removed. It was difficult to remove the bullet safely. We decided to wait for a few days so that the oedema subsided and the exploration of the bullet became easier.

There was confusion regarding the appropriate surgical approach. As the bullet was lodged in the skull base adjacent to the jugulocarotid vessels, a lateral skull base approach like “type-A” infratemporal approach [10] could be adopted. We preferred a less extensive approach to keep the tissue dissection minimum and went for a modified parotidectomy approach. That was sufficient as the position of the bullet was accurately determined preoperatively. There was always the scope to extend the incision in case it was necessary. However, a wider approach from the beginning can be adopted, particularly when the exact position of the foreign body is not known.

## 4. Conclusion

Removal of a foreign body safely from the parapharyngeal space is a challenging task. It is very important to know its exact size, shape, and location, prior to the operation. If the situation permits, a well-planned elective procedure is preferable. A wide exposure is helpful in taking control of the great vessels of the neck. However, a less extensive approach may be sufficient if the accurate location of the foreign body is determined preoperatively.
